# Divergent Temporal Expression of Hyaluronan Metabolizing Enzymes and Receptors with Craniotomy vs. Controlled-Cortical Impact Injury in Rat Brain: A Pilot Study

**DOI:** 10.3389/fneur.2014.00173

**Published:** 2014-09-11

**Authors:** Guoqiang Xing, Ming Ren, Ajay Verma

**Affiliations:** ^1^Department of Neurology, Uniformed Services University of the Health Sciences, Bethesda, MD, USA

**Keywords:** TBI, secondary injury factors, hyaluronic acid, receptor, synthesis, degradation, hyaluronidase, rat brain

## Abstract

Traumatic brain injury (TBI) triggers many secondary changes in tissue biology, which ultimately determine the extent of injury and clinical outcome. Hyaluronan [hyaluronic acid (HA)] is a protective cementing gel present in the intercellular spaces whose degradation has been reported as a causative factor in tissue damage. Yet little is known about the expression and activities of genes involved in HA catabolism after TBI. Young adult male Sprague-Dawley rats were assigned to three groups: naïve control, craniotomy, and controlled-cortical impact-induced TBI (CCI-TBI). Four animals per group were sacrificed at 4 h, 1, 3, and 7 days post-CCI. The mRNA expression of hyaluronan synthases (HAS1-3), hyaluronidases (enzymes for HA degradation, HYAL 1–4, and PH20), and CD44 and RHAMM (membrane receptors for HA signaling and removal) were determined using real-time PCR. Compared to the naïve controls, expression of HAS1 and HAS2 mRNA, but not HAS3 mRNA increased significantly following craniotomy alone and following CCI with differential kinetics. Expression of HAS2 mRNA increased significantly in the ipsilateral brain at 1 and 3 days post-CCI. HYAL1 mRNA expression also increased significantly in the craniotomy group and in the contralateral CCI at 1 and 3 days post-CCI. CD44 mRNA expression increased significantly in the ipsilateral CCI at 4 h, 1, 3, and 7 days post-CCI (up to 25-fold increase). These data suggest a dynamic regulation and role for HA metabolism in secondary responses to TBI.

## Introduction

Traumatic brain injury (TBI) is the leading cause of mortality in children and young adult under 44 years of age in the USA. Brain tissues that are not destroyed immediately following the primary injury may undergo sub-acute injury or delayed death caused by secondarily generated auto-destructive factors (1, 2). Despite extensive research, the mechanism underlying TBI-induced secondary injury remains to be fully elucidated.

Hyaluronan [hyaluronic acid (HA)] is a stable sulfate-free mucopolysaccharide (glycosaminoglycan) containing about 2,500 repeating acetylglucosamine and glucuronic acid disaccharide units and is synthesized by a class of integral membrane proteins, i.e., hyaluronan synthases (HAS1, HAS2, and HAS3). The three HAS genes show distinct patterns of expression during development and their protein products play significantly different roles in the formation of the HA matrix and in response to different stimuli (3–5).

Both HAS1 and HAS2 synthesize high-molecular-weight HA, whereas HAS3 produces lower molecular weight HA (3). The expression of the three HAS isoforms is more prominent in growing cells than in resting cells and is differentially regulated by various stimuli, suggesting distinct functional roles of the three proteins. HAS lengthens hyaluronan by repeatedly adding glucuronic acid and *N*-acetylglucosamine to the nascent polysaccharide. HA is extruded via ABC-transporter through the cell membrane into the extracellular space (6). Hyaluronan forms a protective cementing gel in intercellular spaces throughout the body and acts as a binding and lubricating agent as well as antioxidant (7, 8). Hyaluronan also modulates cell migration, adhesion, wound healing, and tumor invasion (9, 10). The concentration of high-molecular-weight hyaluronan is high in the brains of young rats, but it decreases with aging whereas the low molecular weight hyaluronan increases with aging (11, 12).

As the HAS enzymes are important in cell development and proliferation, they must be strictly regulated. This regulation may occur transcriptionally and post-transcriptionally by naturally occurring anti-sense HAS2 (13–17), by changes in the levels of the sugar substrates needed for HA production, and by modification of the enzymes through HAS dimerization or monoubiquitination (18, 19).

Recent studies in peripheral tissues have implicated a critical role of altered hyaluronan (HA) metabolism in the pathophysiology and healing process of injured tissues. Significantly increased HA production (by 32-fold in the circulation) has been found as a characteristic of patients with acute peripheral lung injury (20) and block HA production by hyaluronan synthase inhibitors effectively suppressed staphylococcal enterotoxin-induced inflammation (21).

The high-molecular-weight hyaluronan is readily degraded into small molecules after tissue injury (22), primarily by increased levels of hyaluronidases (HYALs) and reactive oxygen species (ROS) (23). The degraded hyaluronan fragments play important roles in inflammation, innate immunity, cell proliferation, and wound healing through its antioxidant properties and through interacting with its primary cell surface receptors, CD44, RHAMM, and toll-like receptor 4 (TLR-4) (24–26). The increased fragmentation of HA in the early stages of injury could exert antioxidant effect against ROS and stimulate white blood cells-mediated immune response by up-regulating CD44 (27, 28). Increased levels of hyaluronan and CD44 could also stimulate cell proliferation and migration as found in cancer malignancy (29–31).

Hyaluronidases are a family of lysosomal enzymes that are crucial for the spread of bacterial infections and venoms toxins and the progression of cancer (32–34). Six HYAL genes have been identified [HYAL1, HYAL2, HYAL3, HYAL4, PH20, and HYAL-like pseudogene (HYALP1)] (35–38). Hyal-1 and Hyal-2 are the major mammalian HYALs in somatic tissues, and that they act in concert to degrade high-molecular-weight hyaluronan to the tetrasaccharide (37). HYAL1 is highly expressed in the serum too. HYAL2 enzymes have an acidic pH-optimum with an activity that is considerably lower than for other types of HYALs. HYAL3 is highly expressed in testis and bone marrow but low in other tissues. HYAL4 is expressed in placenta and skeletal muscle and it may form a complex with HYALP1 and PH-20. Human HYALP1 is a pseudogene with mutation in genomic DNA and cDNA (36). HYALs are absent or lowly expressed in normal adult brain. However, injury-induced HYAL expression and HA degradation may alter brain tissue hydration and osmotic balance resulting in edema, and promotes cell proliferation and migration.

Altered HA metabolism has been reported at protracted periods following stroke in human (39) and following middle cerebral artery occlusion (MCAO) in the rat (40). In the human study, the production of total HA and low molecular mass 3–10 disaccharides of HA (o-HA) was increased in post-mortem tissue and in the serum of patients at 1, 3, 7, and 14 days (peaking at 7 days) after ischemic stroke. Hyaluronidase activity was also increased in serum samples (peaking after 3 days) that may underlie the subsequent increase in o-HA (39). Moreover, HA synthases (HAS1 and 2) and HYALs (HYAL1 and 2) protein expression was increased in inflammatory cells from both stroke and peri-infarcted regions of the brain, with HYAL1 upregulated in microvesssels and intracellularly in neurons, while HAS2 became translocated into the nuclei of neurons in peri-infarcted areas (39). And the HA receptor CD44 was increased in infiltrating mononuclear cells in the inflammatory regions. Similar results were found in the rat model of stroke (40).

Hyaluronic acid effects are mediated through two receptors, CD44 and the receptor of HA mediated motility (RHAMM). CD44 is a member of the closely related cell surface glycoproteins [cell adhesion molecules (CAMs)]. CD44 is a 742 amino acid single*-*pass type I membrane protein that is involved in hematopoiesis, lymphocyte activation, and tumor metastasis (41). CD44 mediates both cell–cell and cell–matrix interactions and plays an essential role in cell adhesion and cell migration. CD44 is expressed as multiple isoforms in normal and cancer tissues throughout the body due to alternative splicing events (42, 43). CD44 deficiency is associated with decreased *Cryptococcus neoformans* brain infection (44). When compared to wild type animals, mice deficient in CD44 show significant reduction in ischemic infarct size and in the expression of soluble interleukin*-*1β following transient (30 min ischemia) and permanent (24 h) occlusion of the middle cerebral artery (45). RHAMM, also known as CD168, is a matrix receptor, which is linked to the plasma membrane by a GPI anchor and regulates cell motility. RHAMM is involved in glial cell locomotion and may play a role in the motile behavior of glial cells *in vivo* after CNS injury (46).

So far, no study has examined changes in the hyaluronan pathway after TBI. Considering the importance of hyaluronan metabolism in maintaining the integrity of tissue structure and function and tissue repair, we determined the mRNA expression of hyaluronan receptors (CD44, RHAMM), hyaluronan synthases (HAS1, HAS2, and HAS3), and HYALs (HYAL1, HYAL2, HYAL3, HYAL4, and PH20) in rat brains after controlled-cortical impact-induced TBI (CCI-TBI).

## Materials and Methods

### Animals and controlled-cortical impact-induced TBI

Forty-eight male Sprague-Daley rats (170–200 g) (Taconic Farm, NY, USA) were randomly assigned to three different groups: (1) naïve control; (2) craniotomy (sham CCI); and (3) CCI. Four animals per group were sacrificed at 4 h, 24 h, 3 days and 7 days post-CCI.

For the craniotomy-only and the CCI groups, animals were initially anesthetized with 4% isoflurane in O_2_ with a vented anesthesia chamber connected to an isoflurane scrubber. The rats were mounted in the injury device, secured by ear bars and incisor bar and spontaneously anesthetized with a 1–2% isoflurane in O_2_ via blow-by nose cone connected to a charcoal canister passive isoflurane scavenger. An incision and a 10-mm craniotomy are made over the left primary and secondary motor cortex (bregma 3.70 mm, interaural 12.70 mm). After removal of the bone flap, cortical injury was induced with a CCI device (47), with a penetration depth of 1.5 mm, a velocity of 5 m/s, and a duration of 50 ms over the cortex. The bone scalp was replaced and sealed with dental cement, and the scalp incision was closed with staples following the injury. For the craniotomy alone group, only the cortical injury was excluded from the above animal procedures. Animals were observed after the surgery till they recovered from anesthesia. The animal body temperature was maintained at between 35 and 37°C during the surgery by a warming lamp. All CCI animals looked healthy before and after the CCI injury. All CCI and sham CCI animals recovered from isoflurane anesthesia and became mobile within 5 min after isoflurane discontinuation. Although most CCI animal reassumed some exploratory behavior 30 min after CCI, they did not regain full motor activity till 3 days post-CCI. Animals were sacrificed and transverse (i.e., contralateral and ipsilateral CCI) hemispheres were collected at 4 h, 24 h, 3 days, and 7 days post-CCI (*N* = 4/group/time). For mRNA analysis, the contralateral and ipsilateral hemispheres (coronal sections containing the epicenter of the injury) of the CCI, and the corresponding ipsilateral hemispheres of the naïve and sham rats were separated, rapidly frozen in pre-cooled isopentane (on dry-ice) and stored at −80°C. All animal procedures were approved by the IACUC of the Uniformed Services University of the Health Sciences (USUHS).

### RNA extraction, reverse transcription, and quantitative real-time PCR

Frozen transverse brain hemispheres were homogenized and total RNA was extracted using RNeasy kit (Qiagen, Germany). Total RNA was reverse transcribed into first-strand cDNA in a total volume of 20 μl using the M-MLV reverse transcriptase kit (Promega, Madison, WI, USA). Quantification of mRNA expression was performed in triplicate using the SYBR Green SuperMix (BioRad, CA, USA) in a two-step PCR reaction procedure, performed on the MyiQ single color real-time PCR detection system (BioRad, CA, USA). One microliter cDNA from the RT-reaction was used as the template for quantitative real-time PCR reaction with a final PCR reaction volume of 25 μl, with the 5′ and 3′ gene-specific PCR primer concentrations at 200 nM each. Real-time PCR primers were designed using Primer3 software (Whitehead Institute, MIT, MA, USA) according to the coding sequences of each gene (Table 1). After the initial denaturation at 95°C for 3 min, 40 cycles of primer annealing and elongation were conducted at 60°C for 45 s, followed by denaturation at 95°C for 10 s. Fluorescent emission data were captured, and mRNA levels were quantified using the threshold cycle value (Ct). To compensate for variations in input RNA amounts and efficiency of reverse transcription, qPCR data for mRNA for each sample were normalized by reference to the data obtained for the house keeping beta-actin (GenBank#. BC063166) determined from the same sample. Fold change in mRNA expression was calculated using the equation: fold change = 2^−ΔΔCt^, where ΔCt = target gene Ct – house keeping gene (β-actin) Ct, and ΔΔCt is ΔCt control – ΔCt CCI-TBI (or fold change) = 2^(ΔCT control – ΔCT CCI-TBI)^.

**Table 1:** **Primer sequences for real-time qPCR**.

cDNA bp	Sense primer (5′)	Anti-sense primer (3′)
HAS1 (120)	AGTATACCTCGCGCTCCAGA	ACCACAGGGCGTTGTATAGC
HAS2 (124)	ATAAGCGGTCCTCTGGGAAT	CCCTGTTGGTAAGGTGCCTA
HAS3 (130)	AGCAGCGTGAGGTACTGGAT	AGTCCTCCAGGAACTGCTGA
PH20 (117)	TGGTGAAACAGTTGCTCTGG	GGATTCAGGGTGGTCTTCAA
HYLA1 (107)	ATGACCAGCTAGGGTGGTTG	CTCTTGCACACGGTATCGAA
HYLA2 (107)	AGGCCTGTATCCACGTTTTG	GTTCCACAGCTTCCTTCAGC
HYLA3 (145)	CACCAGATCCTCCACAACCT	GAGGCTGCCTGGTAGACTTG
HYLA4 (133)	ACCCATCAATGGTGGTCTTC	GCGCCAATATTCCCAGTCTA
CD44 (102)	GCTATCTGTGCAGCCAACAA	AAGAGGAGCTGAGGCATTGA
RHAMM (101)	TGCAAAGCCAGTCACTTCTG	GACATTCCTCTCGGAGGTCA

*Oligonucleotide sequences of qPCR primers*.

### Statistical analysis

Data were expressed as mean ± SD. Differences in CD44/HAS/HYAL mRNA expression among the naïve controls, craniotomy, and contralateral and ipsilateral CCI-TBI brains at each time point post-CCI were examined for statistical significance using one-way ANOVA analysis followed by *post hoc* LSD test (two-tailed). A difference with a *p*-value <0.05 was considered statistically significant.

## Results

**Figure 1:**
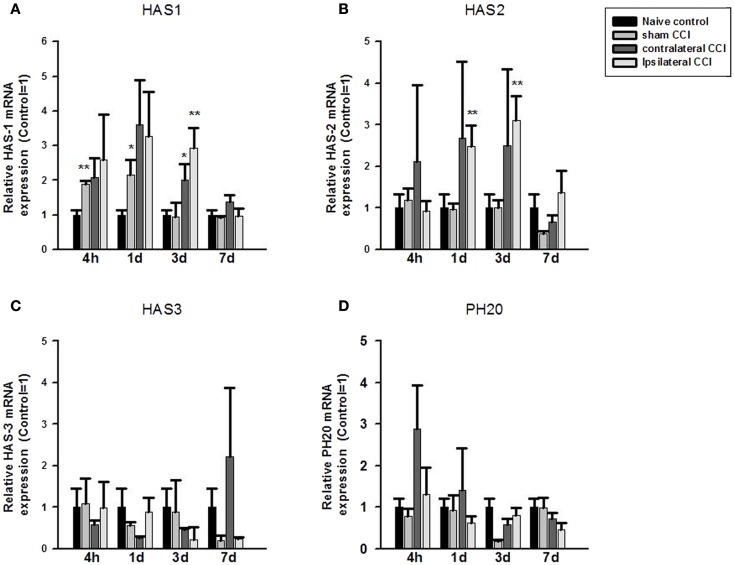
**Quantitative real-time PCR determination of HAS1 (A), HAS2 (B), HAS3 (C), and PH20 (D) mRNA expression in brain homogenates of the control, craniotomy (sham CCI), contralateral CCI, and ipsilateral CCI hemisphere at 4 h, 24 h, 3 days, and 7 days post-CCI**. Results are presented as the fold change relative to the control group (=1). *p < 0.05; **p < 0.01.

Fold change in mRNA expression between the control and CCI/Craniotomy groups was calculated using the qPCR equation: fold change = 2^−ΔΔCt^, where ΔCt = target gene Ct – house keeping gene (β-actin) Ct, and ΔΔCt is ΔCt control – ΔCt CCI-TBI (or fold change). One-way ANOVA showed significant effect of CCI/Craniotomy on HAS1 mRNA expression at 4 h, 24 h, and 3 days after the injury (*p* < 0.01, respectively). *Post hoc* test (two-tailed) showed that compared to that of the naïve control animals, HA synthase 1 (HAS1) mRNA increased significantly (by twofold) in the craniotomy (sham CCI-TBI) at 4 and 24 h post-CCI (*p* < 0.01 and *p* < 0.05, respectively) before returning to the control level 3 days after the craniotomy surgery. HAS1 mRNA expression also increased markedly (two to threefold) in the contralateral and ipsilateral CCI hemispheres at 4, 24, and 72 h post-CCI. And the increase was significance in the contralateral CCI (*p* < 0.05) and ipsilateral CCI (*p* < 0.01) hemispheres at 3 days post-CCI. Thereafter, HAS1 mRNA level returned to control level 7 days post the surgery (Figure 1A).

One-way ANOVA showed significant effect of CCI on HAS2 mRNA expression at 24 h and 3 days post-CCI (*p* < 0.01, respectively). *Post hoc* test showed that compared to the naïve controls, HAS2 mRNA expression increased significantly (>twofold) in the ipsilateral CCI hemisphere at 24 h and 3 days post-CCI (*p* < 0.01, each) (Figure 1B). Thereafter, HAS2 mRNA level returned to basal level 7 days post the injury. Although HAS2 mRNA also increased considerably in the contralateral CCI at 4, 24, and 72 h post the injury, the increase was not significant due to great within-group variation.

No significant effect of craniotomy or CCI-TBI in HAS3 mRNA or in PH20 mRNA expression level was found at anytime after craniotomy and CCI-TBI (Figures 1C,D).

**Figure 2:**
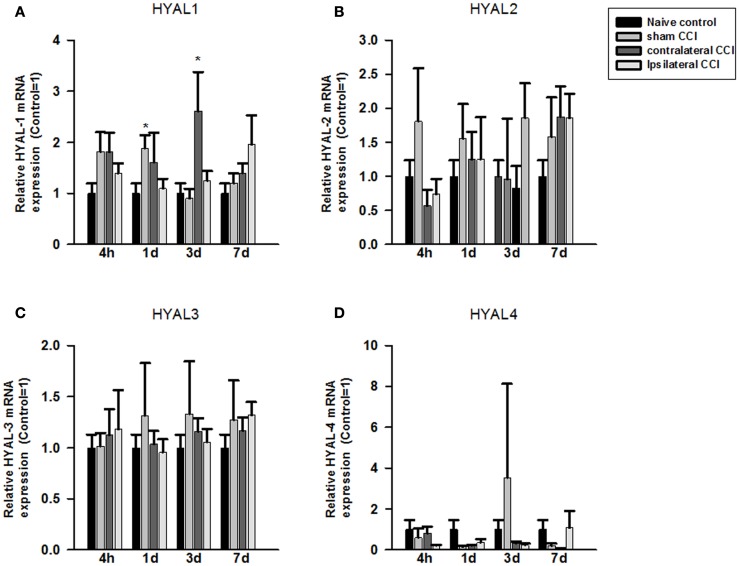
**Quantitative real-time PCR determination of HYAL1 (A), HYAL2 (B), HYAL3 (C), and HYAL4 (D) mRNA expression in brain homogenates of the control, craniotomy (sham CCI), contralateral CCI, and ipsilateral CCI hemisphere at 4 h, 24 h, 3 days, and 7 days post-CCI**. Results are presented as the fold change relative to the control group (=1). *p < 0.05; **p < 0.01

Compared to the naïve groups, hyaluronidase 1 (HYAL1) mRNA expression level increased markedly but non-significantly in the craniotomy group and in the contralateral CCI hemisphere 4 h after craniotomy or CCI (Figure 2A). That increase in HYAL1 mRNA level expression became significant in the craniotomy group at 24 h post the surgery (*p* < 0.05), and in the contralateral CCI hemisphere 72 h after CCI-TBI (*p* < 0.05), respectively (Figure 2A). No significant change in HYAL1 mRNA was found in the ipsilateral CCI hemisphere after CCI-TBI.

No significant change was found in HYAL2, HYAL3, and HYAL4 mRNA expression in the craniotomy and CCI animals after the surgeries (Figures 2B–D).

**Figure 3:**
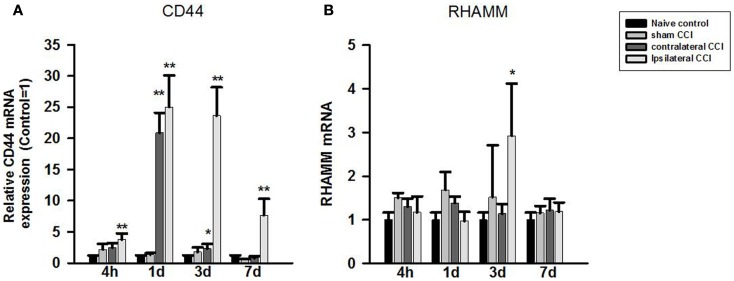
**Quantitative real-time PCR determination of CD44 (A) and RHAMM (B) mRNA expression in brain homogenates of the control, craniotomy (sham CCI), contralateral CCI, and ipsilateral CCI hemisphere at 4 h, 24 h, 3 days, and 7 days post-CCI**. Results are presented as the fold change relative to the control group (=1). *p < 0.05; **p < 0.01.

One-way ANOVA showed significant effect of CCI-TBI on brain CD44 mRNA expression at all four observation time points after CCI. *Post hoc* test showed that compared to that of the naïve controls, CD44 mRNA expression level increased significantly in the ipsilateral CCI hemisphere (>twofold) 4 h, 24 h (by 25-fold), 3 days (23-fold) post-CCI, and 7 days post-CCI (sevenfold) (*p* < 0.01, each) (Figure 3A). CD44 mRNA also increased significantly in the contralateral CCI hemisphere at 24 h (20-fold) and 3 days (twofold) post-CCI (Figure 3A).

In contrast to CD44, RHAMM mRNA level only increased briefly and significantly in the ipsilateral CCI (threefold) (*p* < 0.05) 3 days post-CCI (Figure 3B).

## Discussion

In this study, we found significant sub-acute increases in brain hyaluronic synthases (HAS1 and HAS2) mRNA and in CD44 mRNA expression after CCI-TBI and, to a lesser extent, after craniotomy alone. Although the biological relevance of the increased expression of HAS and CD44 remains to be fully understood, the results suggest that brain HA metabolism could have been altered and may represent a potentially important mechanism of secondary injury and/or repair in TBI. So far there is a lack of information about the regulation of brain HA metabolism after TBI, but recent findings in stroke and in the peripheral tissues could serve as the valuable guide for understanding HA metabolism in injured brain (20, 39).

Altered HA metabolism has been reported at protracted periods following stroke in human (39) and following MCAO in the rat (40). Recent studies in peripheral tissues have implicated a role of altered hyaluronan metabolism in the pathophysiology and healing process of injured peripheral tissues. Significantly increased HA production (by 32-fold in the circulation) has been reported in the acute phase of patients with direct lung injury (20) and blocking HA production by hyaluronan synthase inhibitors effectively suppressed staphylococcal enterotoxin-induced inflammation (21), suggesting increased HA production is potentially involved in the inflammatory or healing process after acute injury or infection.

Studies of organ/tissue during development suggest that intact high-molecular-weight HA is essential for normal vascular development, tissue/organ structure, and functional integrity as absence of HA in HAS2 knockout animal results in reduced HA production and embryonic lethality due to severe cardiac and vascular abnormalities (48). Under physiologic conditions, HA is present as high-molecular-weight (HMW) polymers with an average molecular weigh between 3,000 and 4,000 kDa. HMW HA but not low molecular weight HA is suggested to be able to modulate cytoskeleton regulation, signal transduction, biosynthesis, redox regulation, and protein folding, and act as antioxidant to prevent oxidative stress and cell death after UV-induced injury and to stimulate wound healing (49, 50). However, naïve HA can undergo rapid degradation after tissue injury resulting in accumulation of degraded lower molecular weight species (51, 52) that can induce the expression of a variety of inflammatory factors, including chemokines, cytokines, growth factors, and adhesion molecules in various cell types, indicating an important role of HA in inflammatory processes (26). Studies also showed that degraded hyaluronan products may have biological functions distinct from the native high-molecular-weight polymer. For instance, HA oligomers of 8–16 disaccharides have been found to induce angiogenesis (53, 54), and HA with low to intermediate molecular weight HA (20–450 kDa) have been found to induce the expression of inflammatory genes in macrophages, endothelial cells, eosinophils, and epithelial cells (51, 52, 55–60).

Recent studies showed that treatment with the tetrasaccharide of HA (HA4), significantly enhanced axonal regeneration/sprouting and improved motor function recovery after spinal cord injury in rats and blocked NMDA-induced neuronal cell death *in vitro* (61). Studies also showed that the hyaluronan receptor RHAMM is required for neurite extension and motility in primary neurons and neuronal cell lines (62). And disruption of the hyaluronan-based extracellular matrix in spinal cord promotes astrocyte proliferation (63) whereas HA coating onto the cortical brain after brain damage significantly reduced gliosis, GFAP positive cells, and the thickness of scar formation in the injured brain region at 8 and 12 weeks after the injury in rats (64). Brain tissue scarring (gliosis) is believed to be the major cause of epileptic focus after brain injury, and prevention of scarring could reduce the incidence of seizure.

Several mechanisms have been identified for HA depolymerizing (and thus for CD44 induction) in injured tissues, including HYALs-mediated and ROS-mediated HA depolymerizing processes (65–71). TBI-induced ROS, due to the release of cellular debris of damaged/dead cells (72) and activation of microglia, macrophages, and neutrophils, could directly fragment HA randomly at internal glycoside linkages into smaller fragments (73) and contribute to inflammation (74, 75). ROS-induced Hyal2 expression and the sustained HA fragmentation have been reported in the inflammatory airway lumen of smokers (76). The increased fragmentation of HA in the early stages of injury could exert antioxidant effect against ROS and stimulate white blood cells-mediated immune response by up-regulating CD44 (27, 28).

It has been reported that HYAL2 generates HA fragments of 1–2 kDa (77) that contribute to fibrosis of injured lung tissue (78, 79). Inhibition of hyaluronidase expression and hyaluronan degradation with specific HYAL1, HYAL2, and HYAL3 small interference RNA (siRNAs) significantly reduced CD44 mRNA and protein expression and pro-inflammatory cytokines in mouse synovial fibroblasts after collagen-induced arthritis (80).

In this study, HYAL1 mRNA expression only increased briefly in craniotomy and contralateral CCI-TBI 24 h and 3 days post-CCI. The signaling pathways activating the expression of these genes in TBI are unclear

The marked induction of CD44 mRNA expression after CCI-TBI suggests that CD44 is critically involved in TBI-induced HA catabolism. It is known that CD44-mediated binding, endocytosis, and intracellular degradation of HA are an important mechanism for the removal of local degraded HA within the injured tissues (81–84). The acute and prolonged increase in CD44 mRNA expression is likely paralleled by a change in CD44 protein expression that could reflect an increased production, binding, internalization, and turnover rate of lower molecular weight HA after TBI (85). Hyaluronans bound to CD44 are catabolized in lysosomes (86). Although the mechanism of CD44 turnover in TBI has yet to be fully understood, cytokines, CD44 phosphorylation and induction of alternatively spliced isoforms of CD44 could be involved in the removal of degraded hyaluronan products (39, 40, 64, 85, 87–93).

The increased HA synthases (HAS1 and HAS2) mRNA expression in the acute phase of TBI suggests that synthesis of new HA may be critical for tissue/vascular repair and remodeling after TBI (94, 95). This is supported by the experimental evidence that absence of HA in HAS2 knockout animal causes vascular abnormalities (48), while overexpression of HAS2 promotes neointimal formation after vascular injury (96).

An interaction between fragmented HA and CD44 could stimulate T-cell recruitment (to the sites of inflammation), macrophage activation, neutrophil migration, endothelial cell activation, and the expression of inflammatory genes by activated glial (immune) cells in the injured tissue that could protect against further tissue damage (94, 95, 97). CD44 activation has been shown to protect against hyperoxia-induced lung injury and mortality by a mechanism related to its ability to clear HA from the bronchoalveolar space (98). Failure to clear hyaluronan fragments after the injury may lead to unremitting inflammation. Study show that in the absence of CD44, alveolar macrophages continue to produce chemokines in response to hyaluronan fragments (99).

In this study, we observed parallel change in HAS1, HAS2, and CD44 mRNA expression in the ipsilateral CCI and contralateral CCI hemispheres, and to a lesser extent, in the craniotomy-only group. Although craniotomy has long been used as a control of TBI, it is itself a significant form of injury and can cause morphological damage and functional change as revealed by recent brain imaging and behavioral tests (100). Skull bone removal with high-speed drilling during the craniotomy procedure may not only induce persistent pathological changes in the affected adjacent cortical tissues including altered blood flow, inflammation, and neural cell atrophy but may also cause severe and persistent pain that could trigger HA degradation and systemic inflammatory responses (100). Recent imaging studies have demonstrated a close metabolic connectivity between brain hemispheres and between anatomically separated brain regions (101–105). Although it is not clear yet if this connectivity could have facilitated the transfer of the degraded HA molecules from the ipsilateral hemisphere to the contralateral side, significantly increased HA production (32-fold increase in the circulation) has been reported in patients with acute peripheral lung injury (20). Our observations and other studies also suggest that brain edema can develop and spread to other brain regions rapidly following TBI that may involve altered HA metabolism. The parallel induction of HAS and CD44 mRNA expression between the ipsilateral and contralateral CCI is consistent with the recent reports of rapid and significant global reduction in cerebral blood flow (CBF), cerebral oxygen, and glucose metabolic rate in craniotomized cats (106, 107) and alterations in pyruvate metabolizing enzymes in rats with ipsilateral and contralateral CCI and craniotomy (108). Thus, the synergistic changes in HAS/HYAL/CD44 expression between the ipsilateral, contralateral, and craniotomy could reflect differential brain lesions and blood vessel damage between the ipsilateral/contralateral CCI group and the craniotomy group. Further experiment is needed to firmly establish a close relationship between HA catabolism and HAS/HYAL/CD44 expression in TBI.

There are limitations of the study. The protein expression level for the HAS, HYAL HA-metabolizing isoenzymes, and CD44 receptors were not determined due to the lack of suitable specific antibodies. Nor was the change in HA precursors (i.e., acetylglucosamine and glucuronic acid) and the degraded HA products determined after the CCI-TBI. Further study is warranted for these measurements and to determine whether HA metabolism (synthesis and degradation) is associated with the severity and outcome of patients with severe TBI.

In summary, our study provides preliminary molecular evidence of altered gene expression for HA-metabolizing enzymes and receptors in animal model of TBI. Further study of HA metabolism could help us better understanding of the role of HA in the inflammatory responses, secondary injury, and healing process after TBI.

## Conflict of Interest Statement

The Guest Associate Editor Yumin Zhang declares that despite being affiliated to the same institution as authors Guoqiang Xing, Ming Ren, and Ajay Verma, the review process was handled objectively and no conflict of interest exists. The authors declare that the research was conducted in the absence of any commercial or financial relationships that could be construed as a potential conflict of interest.
